# Stapled bovine pericardial tube graft for infected thoracic aortic reconstruction: Geometric sizing and technical considerations

**DOI:** 10.1016/j.jvscit.2026.102447

**Published:** 2026-08-14

**Authors:** Abdullah G. Alsahwan, Husain Al-Mubarak, Walid A. Alayadhi, Mohammed Al-Omran, Omer Abdulrahim

**Affiliations:** a King Faisal Specialist Hospital and Research Center, Riyadh, Saudi Arabia; b Department of Surgery, King Fahd Hospital of the University, Imam Abdulrahman Bin Faisal University, Al-Khobar, Saudi Arabia; c Division of Vascular and Endovascular Surgery, Department of Surgery, College of Medicine, King Saud University, Riyadh, Saudi Arabia; d Department of Surgery, University of Toronto, Toronto, ON, Canada; e Division of Vascular Surgery, St Michael’s Hospital, Unity Health Toronto, Toronto, ON, Canada; f Li Ka Shing Knowledge Institute, St Michael’s Hospital, Unity Health Toronto, Toronto, ON, Canada

**Keywords:** Bovine pericardium, Endograft infection, Stapled tube graft, Thoracic aortic reconstruction

## Abstract

Aortic reconstruction in infected fields remains surgically challenging because of the risks of persistent infection, graft reinfection, and hemorrhagic complications. Biologic conduits, including bovine pericardial tube grafts, have emerged as attractive alternatives to synthetic grafts in these settings. However, limited data exist regarding stapled graft construction techniques and geometric sizing strategies. A 54-year-old man underwent thoracic endovascular aortic repair for a descending thoracic aortic penetrating ulcer complicated by periaortic hematoma. The postoperative course was complicated by persistent type Ib endoleak requiring thoracic endovascular aortic repair extension, followed by massive hemoptysis and concern for thoracic endograft infection. After staged stabilization and multidisciplinary evaluation, the patient underwent explantation of the infected thoracic endograft with hemiarch and descending thoracic aortic reconstruction using a custom stapled bovine pericardial tube graft. The graft was constructed intraoperatively using bovine pericardial patches and a vascular stapler. Graft sizing was determined using the circumference equation (C = π × D) to achieve accurate luminal diameter and avoid caliber mismatch. Additional overlap margins were incorporated to accommodate stapler application. Technical considerations regarding graft orientation, stapled seam construction, and staple-line exclusion from the final anastomosis are described. Stapled bovine pericardial tube graft reconstruction represents a feasible biologic option for thoracic aortic reconstruction in infected fields. Geometric sizing principles may improve graft calibration and reproducibility during physician-constructed conduit preparation.

Aortic reconstruction in an infected field remains one of the most challenging problems in vascular surgery because of the high risk of persistent infection, graft failure, and reinfection. This problem is particularly relevant after thoracic endovascular aortic repair when an infected endograft or persistent infected periaortic pathology requires definitive source control and durable reconstruction. Biological conduits have gained increasing interest as alternatives to synthetic grafts in these settings because of their lower susceptibility to bacterial colonization and favorable handling characteristics.^1^, ^2^, ^3^, ^4^, ^5^

With the increasing use of thoracic endovascular aortic repair (TEVAR) for complex thoracic aortic pathology, endograft-related infection has emerged as a rare but highly morbid complication, with reported infection rates ranging from approximately 1% to 4% and persistently high mortality.^6^

Bovine pericardial tube grafts have emerged as an attractive biologic alternative for aortic reconstruction in infected fields. In addition to their resistance to infection, bovine pericardial grafts provide favorable pliability, hemostatic properties, and ease of tailoring.^5^^,^^7^^,^^8^ Several techniques for physician-constructed bovine pericardial grafts have been described; however, limited data exist regarding stapled construction methods and geometric sizing strategies.^5^

The purpose of this report is to describe the technical construction of a stapled bovine pericardial tube graft for infected thoracic aortic reconstruction, with particular emphasis on geometric graft sizing principles and operative technical considerations.

## Case presentation

A 54-year-old man with medically managed hypertension and dyslipidemia was referred with complex thoracic aortic pathology. On March 1, 2025, he initially presented to an outside hospital with a descending thoracic aortic penetrating ulcer associated with a periaortic hematoma. TEVAR was performed. The postoperative course was complicated by a type Ib endoleak requiring distal TEVAR extension on March 12, 2025.

On July 6, 2025, the patient presented to our institution with massive hemoptysis estimated at approximately 1 L and hemodynamic instability. Computed tomography angiography demonstrated persistent type Ib endoleak with a large perigraft hematoma concerning for impending rupture. Because of the life-threatening presentation, emergent distal TEVAR extension was performed as a bridge to definitive open reconstruction. Although the extension achieved immediate hemorrhage control, it was not considered definitive because the suspected infected endograft and abnormal periaortic tissue remained in situ.

Blood cultures obtained during the infectious evaluation were negative. However, thoracic endograft infection was suspected on the basis of persistent fever and leukocytosis. Prior antibiotic exposure may have contributed to the negative cultures and did not exclude a localized endograft infection. No fluorodeoxyglucose positron emission tomography scan was performed because the patient had recently undergone multiple thoracic aortic interventions, and postoperative inflammatory changes could have produced increased perigraft uptake and a false-positive result.

After clinical stabilization, the case was reviewed in a multidisciplinary conference involving vascular surgery, cardiac surgery, radiology, and infectious disease teams. Given the concern for thoracic endograft infection and persistent infected aortic pathology, definitive open reconstruction was recommended.

**Fig 1:**
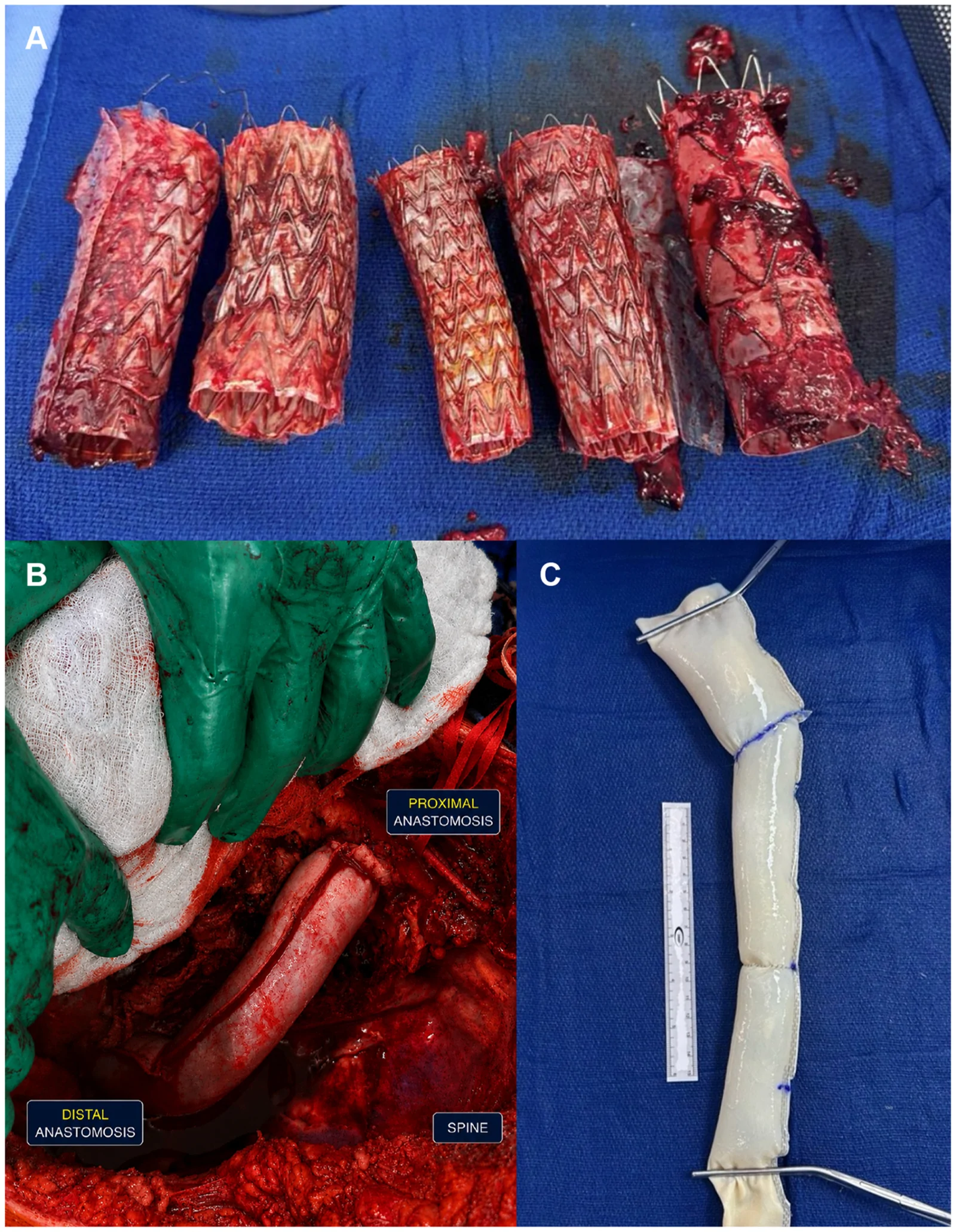
**A,** Explanted infected thoracic endograft components removed during surgery; multiple stent graft segments are shown after extraction. **B,** Intraoperative image demonstrating reconstruction of the hemiarch and thoracic aorta using a bovine pericardial tube graft in situ. **C,** Completed bovine pericardial tube graft fashioned on the back table before implantation.

As the first stage of the planned definitive reconstruction, the patient underwent left carotid-subclavian transposition. Five days later, he underwent the second stage, which included explantation of the infected thoracic endograft covering 17 cm of the aorta, extensive mediastinal debridement, hemiarch replacement, and descending thoracic aortic reconstruction using a custom bovine pericardial tube graft. A cryopreserved aortic allograft was not used because an appropriately sized thoracic allograft was not readily available. The bovine pericardial conduit was selected because it was immediately available and could be tailored intraoperatively to the required diameter and length (Fig 1). Definitive reconstruction was performed through a left-sided trapdoor approach. After systemic heparinization, cardiopulmonary bypass was established using percutaneous right femoral arterial inflow and right femoral venous outflow. A left ventricular vent was placed through the right superior pulmonary vein. The patient was cooled to 18 °C, and hypothermic circulatory arrest was instituted with selective antegrade cerebral perfusion through the innominate artery. Spinal drainage was not used because the reconstructed aortic segment had already been covered by the previous TEVAR.

Postoperatively, the patient received intravenous meropenem and vancomycin for 6 weeks, followed by oral doxycycline and ciprofloxacin for 6 months. He remained in the intensive care unit for 12 days and on the ward for an additional 13 days, for a total postoperative hospital stay of 25 days. Surveillance computed tomography angiography at 1 month and 6 months demonstrated stable conduit caliber and patency without anastomotic complications.

## Technical description

### Operative preparation

The reconstruction was performed using a Supple Peri-Guard bovine pericardial patch (Baxter) measuring 10 × 16 cm, with a manufacturer-reported thickness of 0.25 to 0.75 mm, a 60-mm GIA vascular stapler with Medtronic Tri-Staple technology, and 4-0 polypropylene sutures. The GIA stapler was selected because it simultaneously applied three staggered staple rows and divided the overlapping patch edges, creating a straight longitudinal seam while removing excess material. Its 60-mm length also facilitated rapid and reproducible construction of the long conduit. In contrast, a transverse anastomosis stapler would have required a separate step to trim the excess patch.

### Graft sizing

Accurate graft sizing is essential to maintain luminal diameter and avoid graft kinking, stenosis, or caliber mismatch. The target graft diameter was determined preoperatively using computed tomography angiography measurements obtained at the planned anastomotic site.

In this case, the desired graft diameter was approximately 32 mm. To construct the tube graft, the required width of the bovine pericardial patch was calculated using the circumference equation:

C=π×D

**Fig 2:**
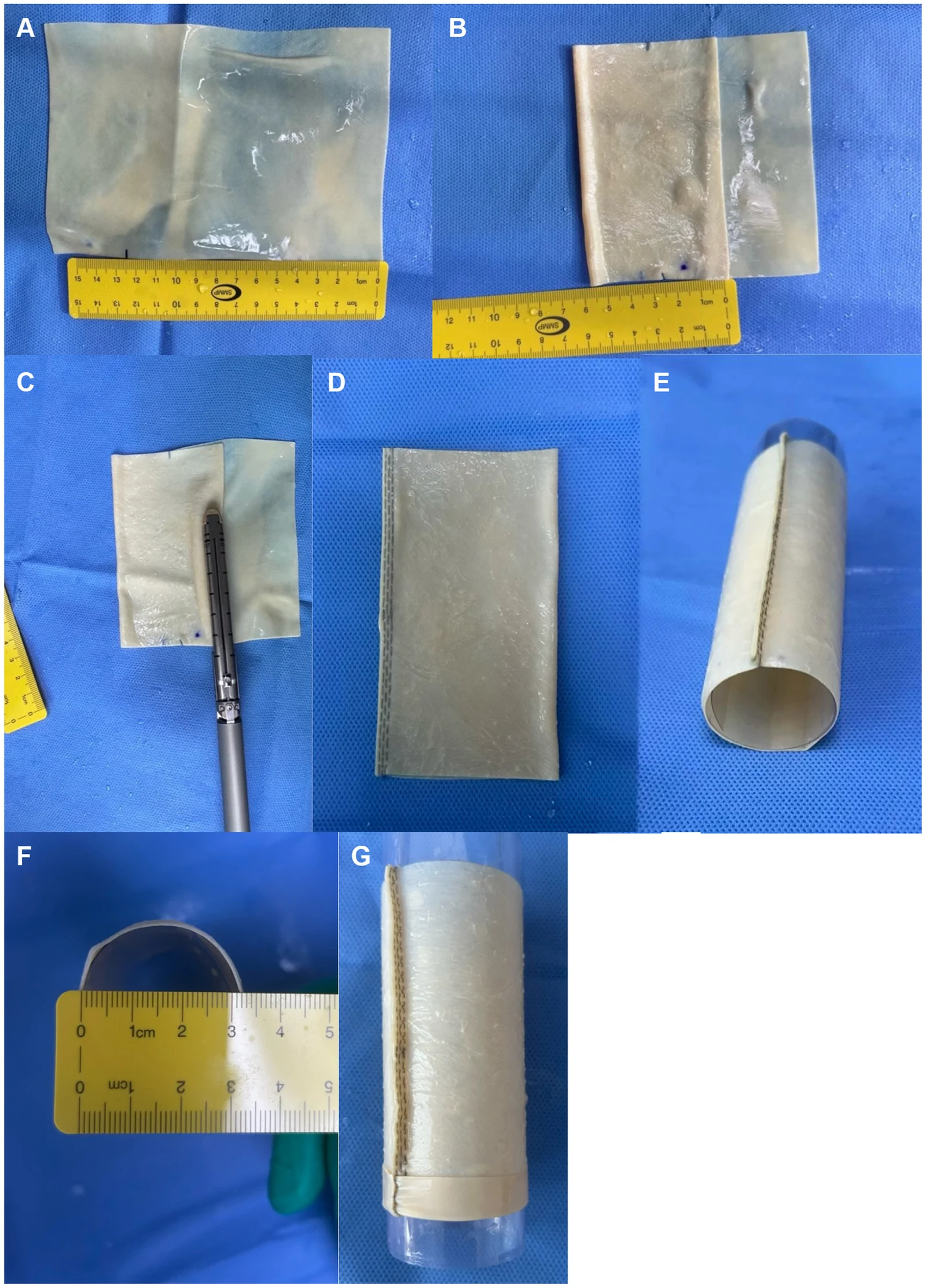
**A,** The required graft circumference was calculated to be 10.48 cm, and an additional 2 cm was added (1 cm per edge) to accommodate the stapler application site. **B,** The bovine pericardial patch was rolled into a tubular configuration, and a small vascular clip was applied at the midpoint to maintain alignment and facilitate graft construction. **C-E,** A vascular stapler was used to construct the bovine pericardial tube graft after rolling the patch into a tubular configuration. **F,** The desired graft diameter of 32 mm was successfully achieved. **G,** The graft edge was everted to exclude the stapled edge from the final suture line during anastomosis.

For a target diameter of 32 mm, the calculated circumference was approximately 100.5 mm. An additional 10 mm was added to each edge to provide adequate overlap for stapler application (Fig 2, *A*).

### Tube graft construction

The smooth surface of the bovine pericardial patch was oriented toward the graft lumen to potentially reduce thrombogenicity. The patch was rolled into a cylindrical configuration, and small vascular clips were temporarily applied to maintain alignment and uniform tension during construction (Fig 2, *B*). Care was taken to avoid twisting, redundancy, or excessive overlap of the patch edges.

The longitudinal seam was created using two to three sequential vascular stapler loads, producing a hemostatic tube graft of uniform diameter (Fig 2, *C-F*). The reconstructed thoracic aortic segment measured approximately 16 cm in length. When additional graft length was required, a second bovine pericardial tube graft was constructed and anastomosed in an end-to-end fashion using a running 4-0 polypropylene suture.

### Staple-line considerations

The graft was constructed using multiple firings of a 60-mm stapler, producing three parallel staple rows. Each firing was carefully aligned with the preceding staple rows to maintain a straight, continuous seam, as misalignment could create a focal weak point and increase the risk of seam disruption or graft failure. During implantation, the stapled seam was oriented anteriorly to allow direct inspection and facilitate reinforcement or repair should bleeding or any seam-related complication occur after completion of the arterial anastomosis. The stapled seam was excluded from the anastomosis to reduce direct tension and facilitate hemostasis (Fig 2, *G*).

## Discussion

Infected thoracic endografts remain among the most challenging conditions encountered in vascular and aortic surgery because of the combined risks of hemorrhage, sepsis, and graft reinfection. Successful treatment requires aggressive debridement, durable reconstruction, and careful selection of conduit material.^1^^,^^6^^,^^9^, ^10^, ^11^, ^12^

Although synthetic grafts remain widely used, their application in infected fields is associated with persistent concerns regarding bacterial colonization and recurrent infection.^1^, ^2^, ^3^ Alternative biologic conduits, including autologous femoral vein grafts and cryopreserved allografts, have demonstrated favorable resistance to infection but may be limited by availability, operative complexity, donor-site morbidity, and long-term durability.^4^^,^^13^

Bovine pericardial tube grafts have emerged as an attractive biologic alternative for reconstruction in contaminated fields. Their pliability, hemostatic characteristics, and resistance to infection make them an attractive option for complex aortic reconstruction.^4^^,^^5^^,^^7^^,^^8^ In addition, bovine pericardial conduits can be tailored intraoperatively to accommodate variable anatomy and facilitate individualized reconstruction.

**Fig 3:**
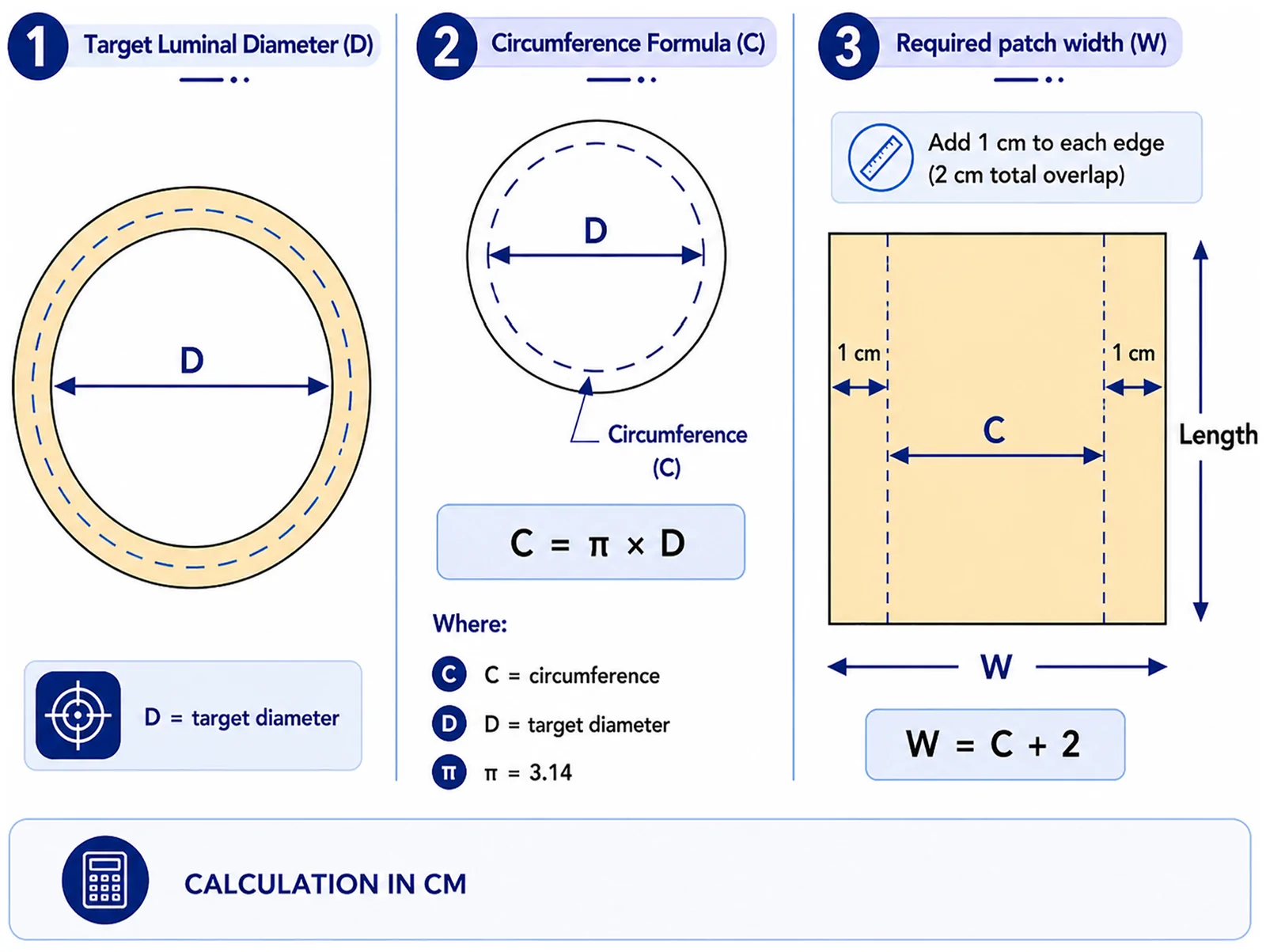
Circumference-based graft sizing calculation for bovine pericardial tube graft construction.

Several methods for constructing bovine pericardial tube grafts have been described.^5^ In the present case, stapled construction facilitated rapid intraoperative graft preparation while maintaining a uniform cylindrical configuration. The use of geometric sizing based on the circumference equation provided accurate graft diameter estimation and helped minimize caliber mismatch. This circumference-based sizing method represents a practical technical contribution because it translates preoperative target diameter measurements into a reproducible patch width for intraoperative graft construction (Fig 3).

Long-term durability data for physician-constructed bovine pericardial tube grafts remain limited, particularly in thoracic aortic reconstruction.^4^^,^^5^ Nevertheless, recent multicenter experiences have reported encouraging early and midterm outcomes in infected aortic pathology.^4^^,^^5^ Weiss et al^5^ reported encouraging outcomes using physician-constructed bovine pericardial tube grafts for infected aortic pathology, supporting the feasibility of this approach in complex infected reconstructions.

This report has several limitations, including the single-case design, limited follow-up duration, and lack of comparative analysis with alternative biologic conduits. Additional long-term studies are required to better define durability and late outcomes of stapled bovine pericardial tube graft reconstruction in thoracic aortic infection.

This case demonstrates that stapled bovine pericardial tube graft reconstruction, combined with geometric circumference-based sizing principles, may represent a feasible and reproducible option for complex thoracic aortic reconstruction in infected fields when conventional conduits are less desirable or unavailable. Written informed consent was obtained from the patient for publication of this case report.

## Funding

None.

## Disclosures

None.
